# Treatment of tumor thrombus in the superior mesenteric vein due to advanced colon cancer with complete surgical resection and chemotherapy: a case report

**DOI:** 10.1186/s40792-020-01091-6

**Published:** 2020-12-14

**Authors:** Yoshitsugu Yanagida, Takahiro Amano, Ryuji Akai, Akira Toyoshima, Jotaro Kobayashi, Takuya Hashimoto, Eiji Sunami, Toshio Kumasaka, Shin Sasaki

**Affiliations:** 1 Department of Coloproctological Surgery, Japanese Red Cross Medical Center, 4-1-22, Hiroo, Shibuya-ku, Tokyo 150-8935 Japan; 2 Department of Gastroenterological Surgery, Cancer Institute Hospital, Japanese Foundation for Cancer Research, 3-8-31 Ariake, Koto-ku, Tokyo 135-8550 Japan; 3 Department of Cardiovascular Surgery, Japanese Red Cross Medical Center, 4-1-22, Hiroo, Shibuya-ku, Tokyo 150-8935 Japan; 4 Department of Hepato-Biliary-Pancreatic and Transplantation Surgery, Japanese Red Cross Medical Center, 4-1-22 Hiroo , Shibuya-ku, Tokyo 150-8935 Japan; 5 Department of Surgery, Kyorin University Hospital, 6-20-2, Shinkawa, Mitaka City, Tokyo 181-8611 Japan; 6 Department of Pathology, Japanese Red Cross Medical Center, 4-1-22, Hiroo, Shibuya-ku, Tokyo 150-8953 Japan

**Keywords:** Colorectal cancer, Adjuvant chemotherapy, Tumor thrombus, Thrombectomy

## Abstract

**Background:**

Tumor thrombus in the superior mesenteric vein secondary to colon cancer is rare. We report a case of tumor thrombus in the superior mesenteric vein and liver metastasis due to advanced colon cancer that was treated with chemotherapy and complete surgical resection.

**Case presentation:**

A 72-year-old man after transverse colectomy with lymph node dissection for advanced colon cancer was diagnosed with tumor thrombus in the superior mesenteric vein and liver metastasis. He underwent adjuvant chemotherapy and had complete surgical tumor resection involving tumor thrombectomy and hepatectomy. There has been no recurrence at 36 months after surgery.

**Conclusion:**

Herein, we report a rare case of tumor thrombus in the superior mesenteric vein related to advanced colon cancer. The combination of chemotherapy and complete surgical tumor resection may provide long-term survival.

## Background

Colon cancer is likely to metastasize to other organs synchronously or metachronously. However, tumor thrombus in the superior mesenteric vein (SMV) is a rare pattern of metastatic recurrence [1–3], and the strategies for treatment of such a metastatic lesion and its prognosis are unclear. According to recent reports, following complete surgical tumor resection and chemotherapy, some patients achieved long-term survival [4]. Herein, we report a rare case of tumor thrombus in the SMV and liver metastasis related to advanced colon cancer that was treated with chemotherapy and complete surgical tumor resection involving tumor thrombectomy and hepatectomy.

## Case presentation

A 72-year-old man with a history of chronic hepatitis C presented to our department with a high carcinoembryonic antigen (CEA) level (26.0 ng/mL) on his blood test results. He had a past history of a duodenal ulcer.

**Fig. 1:**
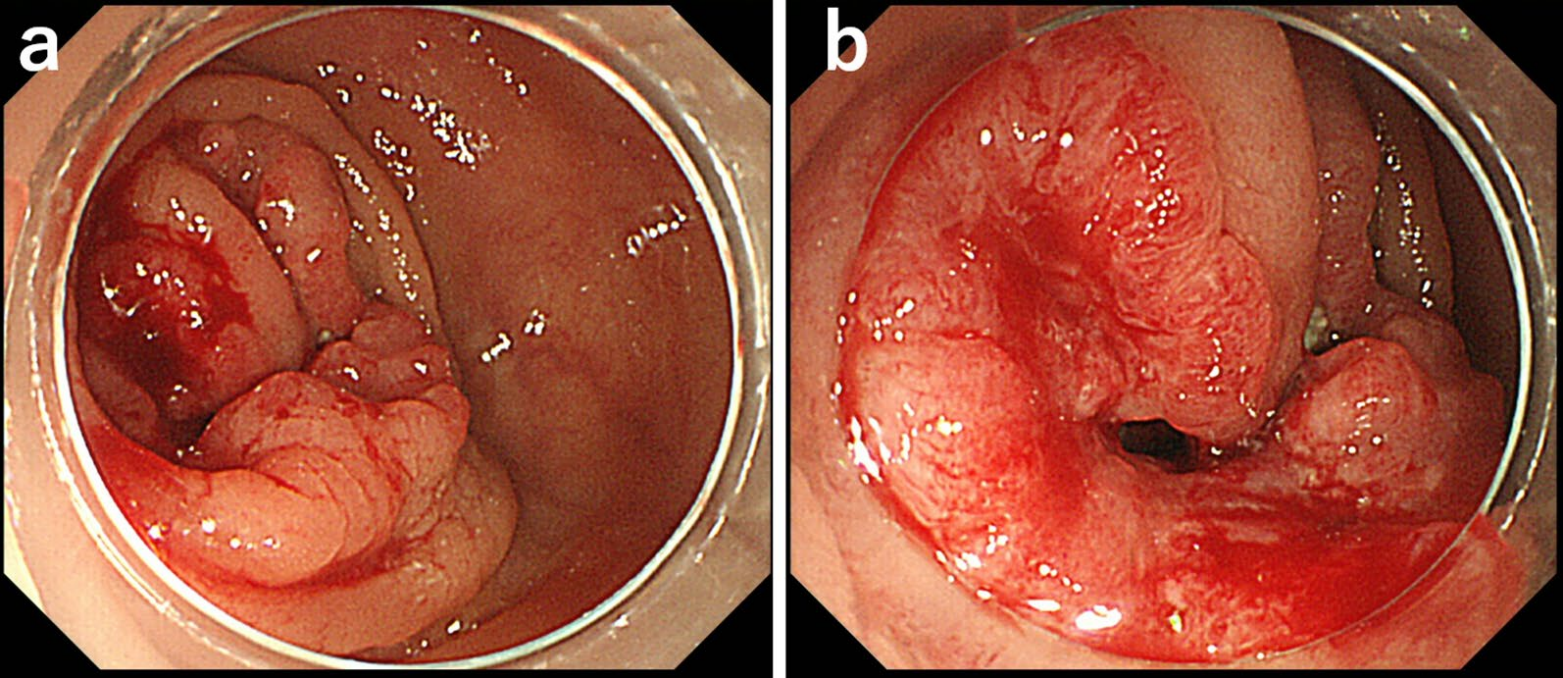
Colonoscopy image. Colonoscopy revealing a mass in the transverse colon (**a**). An obvious random wall was not identified. The colon was almost obstructed by the tumor, and the scope could not pass the lesion, which we considered as a type 3 tumor (**b**)

**Fig. 2:**
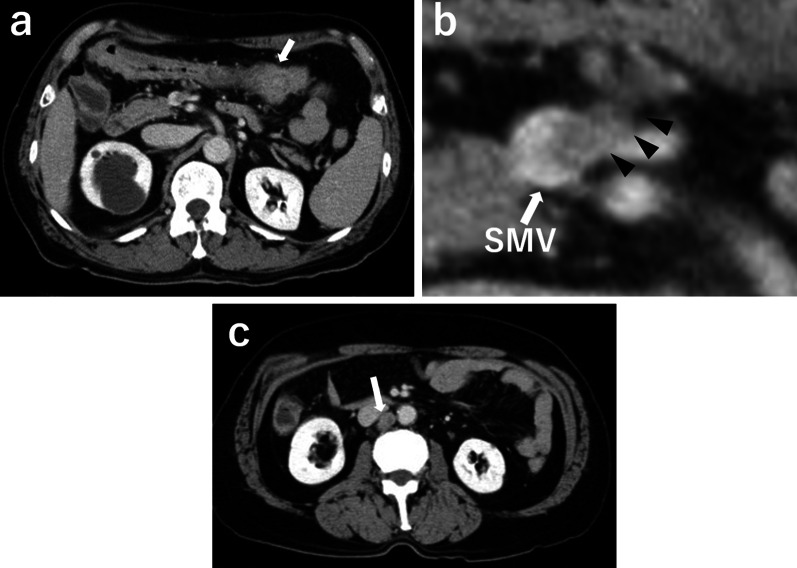
Computed tomography (CT) images. Abdominal CT showing an enhanced mass in the transverse colon (**a**, arrow), with mesenteric lymph node metastasis invading the superior mesenteric vein (**b** enlarged image of **a**, arrowhead) and paraaortic lymph node metastasis (**c**, arrow). SMV, superior mesenteric vein

His other laboratory results were as follows: white blood cell count, 5.0 × 10^3^/µL; hemoglobin, 10.6 g/dL; platelet count, 13.6 × 10^4^/µL; aspartate aminotransferase(AST)/alanine aminotransferase (ALT) levels, 24/14 IU/L; plasma sodium level, 139.0 mEq/L; plasma potassium level, 4.1 mEq/L; and cancer antigen 19-9 (CA19-9) level, 29 U/mL. Colonoscopy revealed a type 3 tumor in the transverse colon, and the endoscope could not progress past the lesion (Fig. 1). Abdominal computed tomography (CT) showed a tumor in the transverse colon with mesenteric and paraaortic lymph node metastases and invading the SMV (Fig. 2). He was diagnosed with advanced transverse colon cancer (stage IVA, TNM classification).

**Fig. 3:**
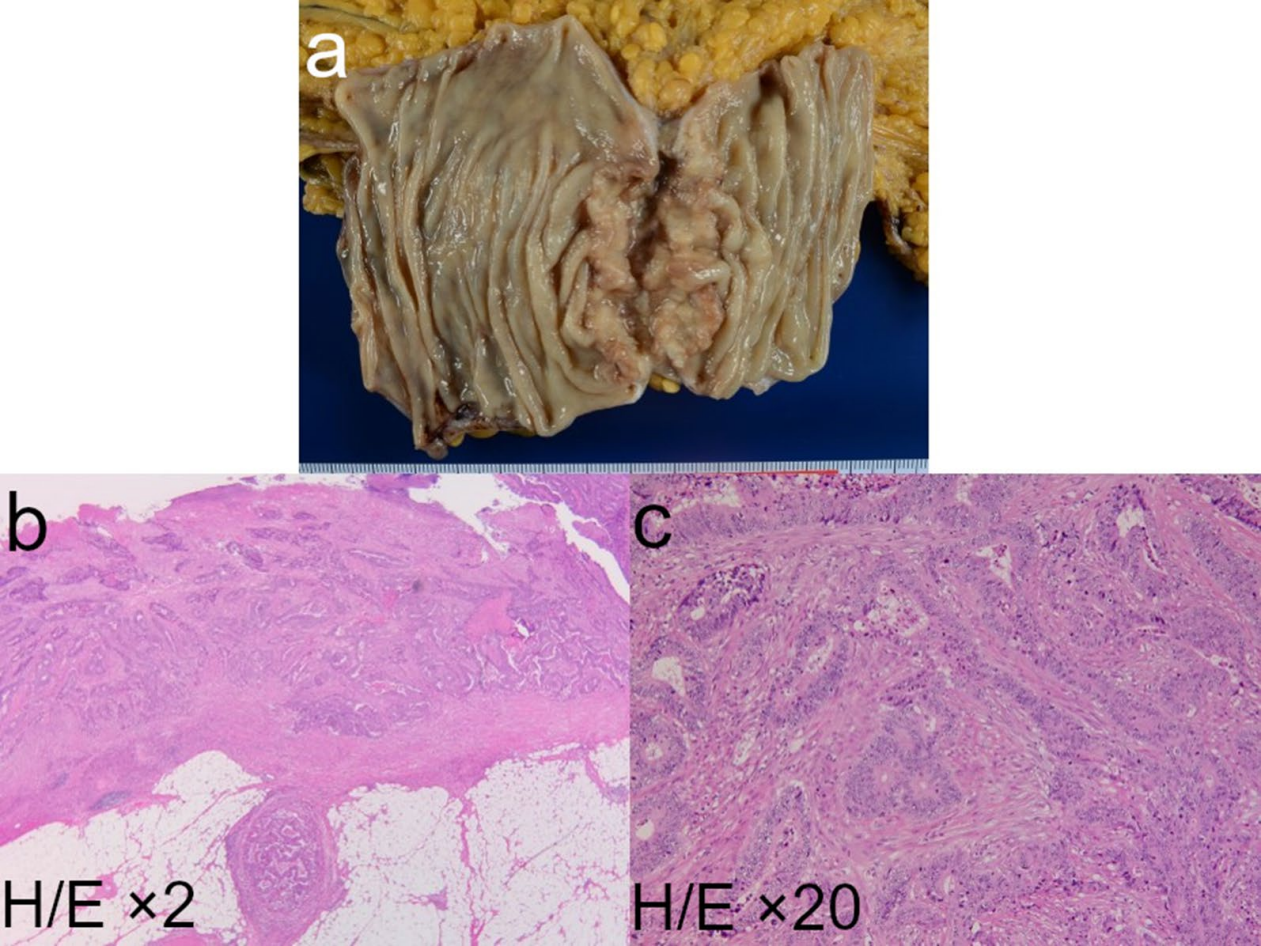
Pathological images of transverse colectomy. Macroscopic (**a**) and microscopic (**b** and **c**) images of the resected specimen. The advanced tumor was located in the transverse colon, which obstructed the colon (**a**). Microscopically, the colon cancer was a moderately differentiated adenocarcinoma that reached the subserosal layer with lymphatic/venous duct involvement (**b** and **c**). *H/E* hematoxylin and eosin staining

**Fig. 4:**
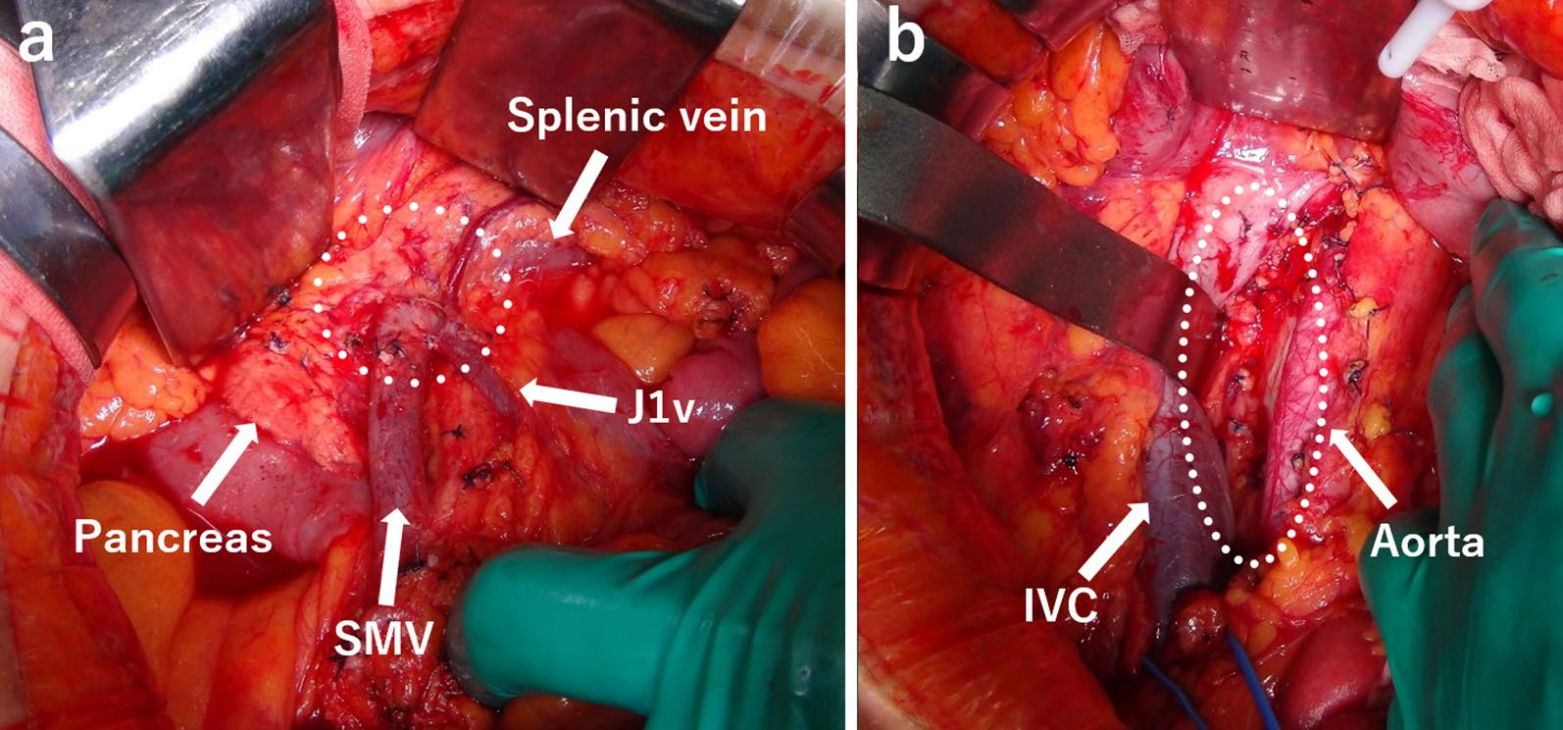
Mesenteric and paraaortic lymph node dissection. Lymph node dissection involving the mesenteric lymph node around the superior mesenteric vein (white dotted line) and paraaortic lymph node (**b** white dotted line). *SMV* superior mesenteric vein, *J1v* first jejunal vein, *IVC* inferior vena cava

**Fig. 5:**
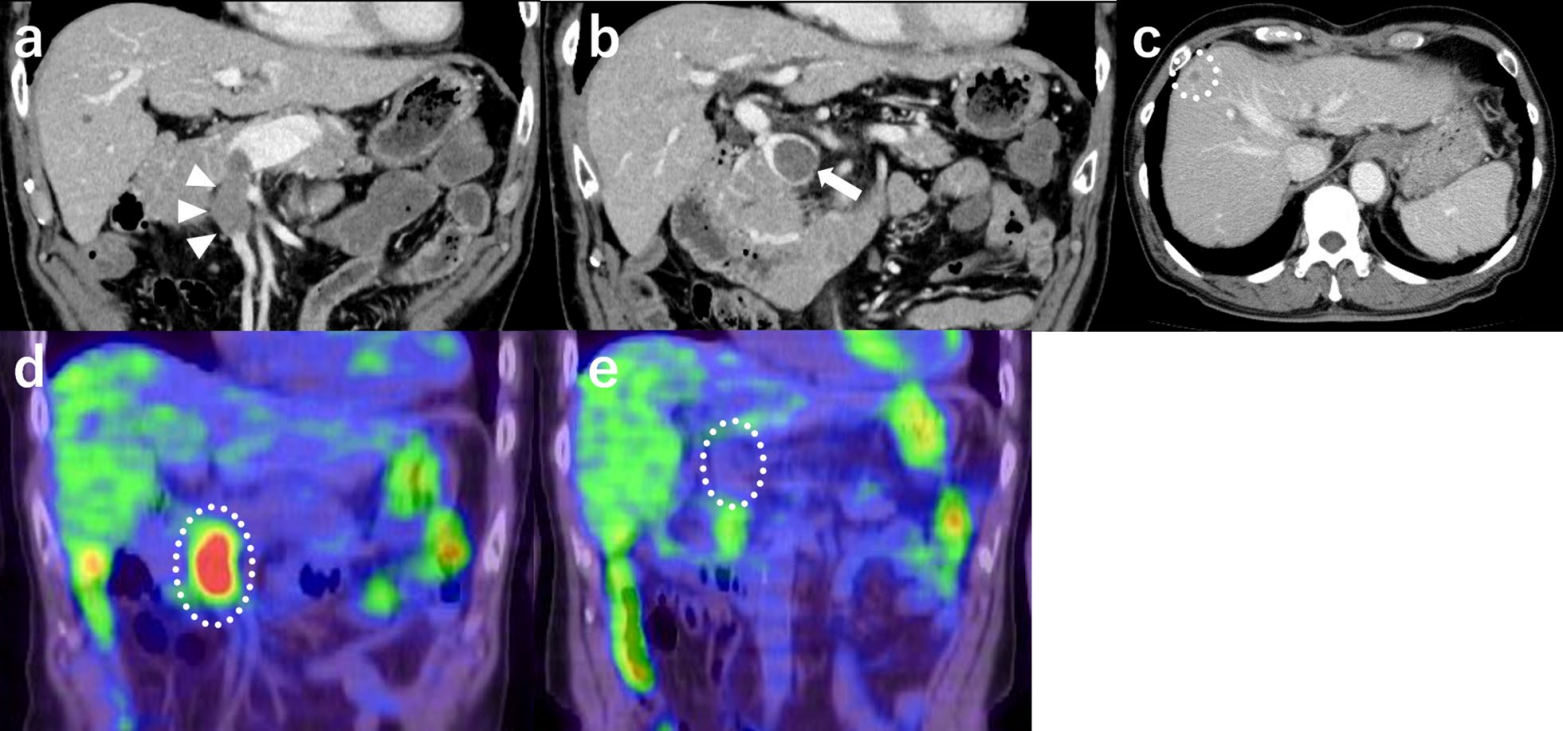
Computed tomography (CT) and positron emission tomography (PET)–CT images. CT showing tumor thrombosis in the superior mesenteric vein (SMV; **a** arrowhead), portal vein thrombosis (**b** arrow), and liver metastasis (**c** white dotted line). On PET–CT, the tumor thrombus in the SMV demonstrated fludeoxyglucose uptake (**d** white dotted line), but the portal vein thrombus did not (**e** white dotted line)

**Fig. 6:**
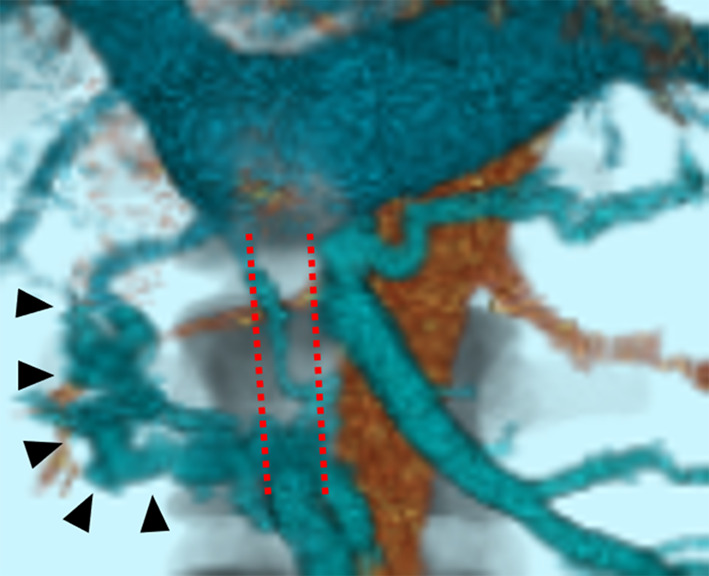
Computed tomography image. The superior mesenteric vein is completely obstructed by the tumor thrombus (red dotted line) and collateral circulations have developed (arrowhead)

**Fig. 7:**
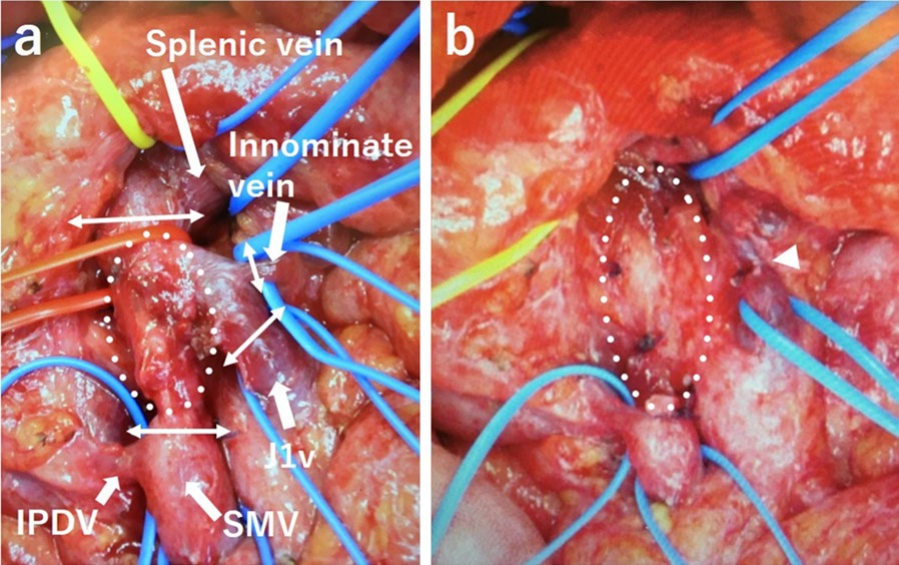
Tumor thrombectomy. We confirmed the range of tumor thrombus in the superior mesenteric vein (**a** white dotted line). We completely resected it (**a** white double-head arrow) with a reanastomosis between J1v and innominate vein from the duodenum (**b** arrowhead). *SMV* superior mesenteric vein, *IPDV* inferior pancreatico-duodenal vein, *J1v* first jejunal vein

**Fig. 8:**
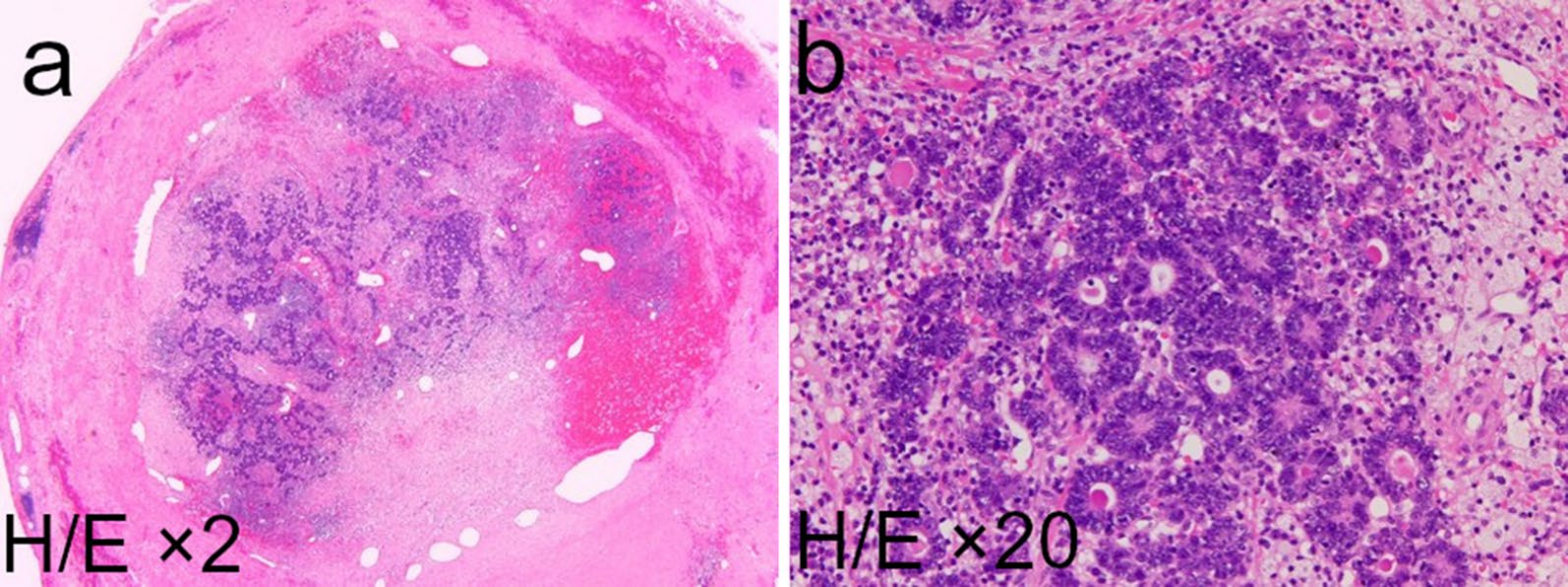
Pathological images of tumor thrombus in the SMV. Tumor thrombus existed inside the SMV and did not invade out of the SMV. *H/E* hematoxylin and eosin staining

**Fig. 9:**
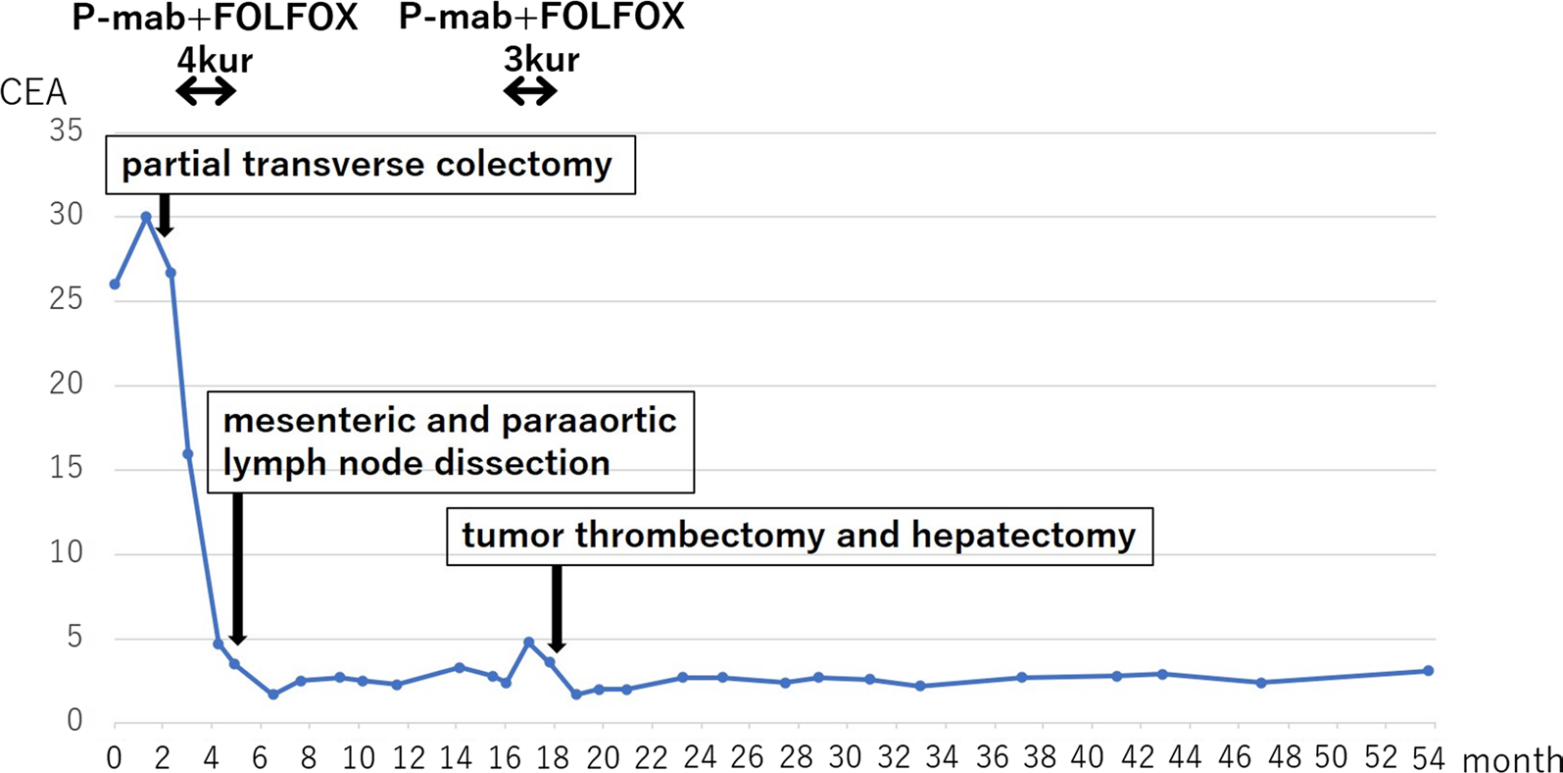
Summary of the treatment. Trends in serum carcinoembryonic antigen (CEA) levels are indicated by solid lines. CEA levels decreased promptly after the first surgery and postoperative chemotherapy, and their normalization continued for 36 months after the last surgery

First, the patient underwent a partial transverse colectomy because the colon was obstructed by the tumor. The procedure time was 239 min, and blood loss was 130 mL. Pathological examination showed that the tumor was a moderately differentiated adenocarcinoma of the transverse colon, encroaching the subserosal layer, with adjacent lymph node metastasis (3/4) and lymphatic/venous duct involvement (Fig. 3). A mutation in the *K-RAS* gene was not detected. PET–CT after four cycles of chemotherapy (panitumumab + FOLFOX) for lymph node metastasis showed no new metastatic lesions. Following this, the patient underwent mesenteric and paraaortic lymph node dissection with extended right hemicolectomy (Fig. 4). The procedure time was 564 min, and blood loss was 310 mL. No residual tumor in the colon and no lymph node metastasis around the SMV (0/41), except for one paraaortic lymph node metastasis (1/33), were pathologically detected. We could not perform adjuvant chemotherapy because the patient presented with ascites, which was most likely related to his chronic hepatitis C and operative invasiveness, and required readmission and paracentesis. After 10 months, CT and PET–CT showed tumor thrombus in the SMV, portal vein thrombus, and liver metastasis without ascites (Fig. 5). We started him on an oral anticoagulant drug and performed three cycles of chemotherapy (panitumumab + FOLFOX) and confirmed a reduction of tumor thrombus in the SMV after chemotherapy. We also confirmed the presence of collateral circulation, which permitted us to resect the SMV itself without revascularization (Fig. 6). Finally, the patient underwent tumor thrombectomy, removal of the portal vein thrombus, and partial hepatectomy. We carefully evaluated the range of tumor thrombus and determined the excision range of the SMV, confirming negative surgical margins during surgery (Fig. 7). The procedure time was 566 min, and blood loss was 1110 mL. Tumor thrombus in the SMV and liver metastasis with negative surgical margins and the absence of tumor cells in the portal vein thrombus were pathologically confirmed (R0 resection). Pathological findings are shown in Fig. 8, which indicates that the tumor thrombus existed not outside but inside the wall of the SMV. The patient was considered for adjuvant chemotherapy, but it was not possible because he presented with temporal pancytopenia, probably owing to chronic hepatitis C and operative invasiveness. Thirty-six months after the last surgery, no recurrence was detected. The patient’s clinical course after diagnosis is shown in Fig. 9.

## Discussion

Venous tumor thrombus occasionally accompanies some carcinomas, such as inferior vena cava thrombus of renal cell carcinoma [5] and portal vein thrombus of hepatocellular carcinoma [6]. However, venous tumor thrombus related to colon cancer is quite rare [1]. Sato reported that venous tumor thrombus was detected in only 3 (1.7%) out of 176 patients with advanced colon cancer [2], and Tada reported that the incidence of venous tumor thrombus related to advanced colon cancer was approximately 2.8% [3].

According to Otani’s review, the invaded vein was dependent on the primary tumor site; ascending and transverse colon cancer invaded the SMV, whereas descending, sigmoid colon, and rectal cancer invaded the inferior mesenteric vein (IMV) [4]. In our case, the tumor thrombus developed in the SMV as the primary lesion was in the transverse colon and drained into the SMV through the middle colic vein. Otani et al. also reported that the pathological type of more than half of the cases with venous tumor thrombus was identified as moderately differentiated adenocarcinoma [4]. Furthermore, moderately differentiated adenocarcinoma is likely to have a higher malignant potential than well-differentiated adenocarcinoma [7–9] and was associated with higher serum CEA levels [10, 11]. In our case, the pathological type of the primary lesion and the tumor thrombus was a moderately differentiated adenocarcinoma. In addition, the patient’s tumor was found because of the high serum CEA level.

Enhanced CT is useful for detecting venous tumor thrombus. We were able to detect tumor thrombus in the SMV using enhanced CT, which also indicated the presence of portal vein thrombus. Both tumor thrombus and blood clot thrombus appear as low-attenuation areas on CT. Recently, PET–CT has been regarded as useful in detecting venous tumor thrombus through intense radiotracer accumulation and helps distinguish tumor thrombus from blood clot thrombus [12]. In our case, PET–CT showed the presence of a tumor thrombus in the SMV and portal vein thrombus; the former demonstrated fludeoxyglucose uptake, but the latter did not.

Fujii reported that the precise range of tumor thrombus and presence of collateral circulation should be evaluated before performing tumor thrombectomy [13]. In our case, CT showed occlusion of the SMV by the tumor thrombus and the presence of collateral circulation, enabling us to excise the SMV without revascularization.

Table 1 shows the reported cases of colon cancer accompanied with tumor thrombus in the SMV [14–22]. Ten cases have been reported, including our case. The ascending colon was the most common site of tumor thrombus in the SMV, and the most common histological type was moderately differentiated carcinoma, similar to our case.

**Table 1:** Reported cases of colon cancer accompanied with tumor thrombus in the superior mesenteric veins

No.	First author	Year	Age/sex	Location of primary lesion	Histological type	ly/v	Complete resection	Adjuvant chemotherapy	Recurrence	Prognosis
1	Kawashima [14]	2007	78/F	A	Poorly differentiated adenocarcinoma	ly3/v3	Done	5-FU/LV	Liver(4 M)	Dead(5 M)
2	Kanzaki [15]	2009	68/M	T	Moderately differentiated adenocarcinoma	ly2/v3	Done	FOLFOX4, UFT/LV	(–)	Alive(24 M)
3	Yamagami [16]	2009	66/F	A	Moderately differentiated adenocarcinoma	ly2/v3	Done	FOLFIRI	(–)	Alive(22 M)
4	Kamata [17]	2015	70/F	A	Well-differentiated adenocarcinoma	ly1/v1	Done	FOLFOX4	(–)	Alive(9 M)
5	Tajima [18]	2016	60/F	A	Moderately differentiated adenocarcinoma	ly3/v3	no	(–)	Liver, dissemination	Dead(21 M)
6	Akabane [19]	2018	48/F	A	Moderately differentiated adenocarcinoma	ly0/v2	Done	CapeOx	(–)	Alive(17 M)
7	Kim SE [20]	2019	46/F	A	Poorly differentiated adenocarcinoma	N.D	Done	FOLFIRI + Bev	(–)	Alive(12 M)
8	Greally M [21]	2019	54/F	T	Mucinous adenocarcinoma	N.D	Done	FOLFIRI	(–)	Alive(18 M)
9	Fujii [22]	2020	82/M	A	Moderately differentiated adenocarcinoma	N.D	Done	(–)	Liver (6 M)	Dead(8 M)
10	Our case		72/M	T	Moderately differentiated adenocarcinoma	ly1/v1	Done	(–)	(–)	Alive(36 M)

*M* male, *F* female, *A* ascending colon, *T* transverse colon, *5-FU* fluorouracil, *LV* leucovorin, *UFT* tegafur–uracil, *Bev* bevacizumab, *FOLFOX* oxaliplatin/5-FU/leucovorin, *FOLFIRI* irinotecan/5-FU/leucovorin, *CapeOx* capecitabine/oxaliplatin

The combination of complete surgical tumor resection and chemotherapy is important for the treatment of venous tumor thrombus. In our case, we performed three operations in total for the primary lesion and metastatic lesions, and eventually, we were able to completely resect not only the tumor thrombus, but also the liver metastasis. Chemotherapy, particularly adjuvant chemotherapy, also plays an important role in treating tumor thrombus even if complete surgical tumor resection is performed considering the aggressiveness of advanced colon cancer with venous tumor thrombus, which may have a higher risk of metastasis or recurrence [4, 14]. In our case, the patient underwent only preoperative chemotherapy for tumor thrombus, and we were able to evaluate the resectability of the tumor thrombus. The patient did not undergo adjuvant chemotherapy because of pancytopenia, probably secondary to chronic hepatitis C and operative invasiveness.

Recently, Arnold et al. reported that chemotherapy plus anti-epidermal growth factor receptor (EGFR) antibody therapy was not significantly beneficial for patients with right-sided colon cancer and KRAS wild-type for overall survival and progression-free survival [23]; similar suggestions were echoed in the Japanese Society for Cancer of the Colon and Rectum guidelines 2019 for the treatment of colorectal cancer. In this case, we used an anti-EGFR antibody drug because Arnold’s report had not yet been published. It has been reported that anti-EGFR antibody drugs have an advantage in response rate in comparison with bevacizumab [24–26]. In addition, Salvatore et al. reported that anti-EGFR antibody drugs might be associated with a higher chance of early tumor shrinkage and a better depth of response [27]. We then selected the anti-EGFR antibody drug to obtain the best response rate and to be able to perform conversion surgery.

The prognosis of patients with venous tumor thrombus related to colon cancer is unclear. Akabane reported 12 cases of colon cancer with tumor thrombus in the SMV or IMV, and metastatic recurrence occurred in 5 of 12 cases after surgical resection [19]. As venous tumor thrombus may indicate an aggressive character of cancer, the recurrence rate may be higher and the prognosis may be worse. However, some patients have a relatively good prognosis after complete surgical tumor resection and chemotherapy [19–21]. In these cases, the patients underwent R0 resection and achieved long-term survival, which is similar to our case. According to a meta-analysis by Otani, 11 of 43 patients with venous tumor thrombus related to colon cancer survived for more than 2 years, although 5 of the patients had liver metastasis. Furthermore, the mean survival time of patients with liver metastasis was 22.5 months [4]. In our case, we first administered preoperative chemotherapy and then performed complete surgical tumor resection involving tumor thrombectomy and hepatectomy. We then achieved R0 resection, and the patient obtained 36 months of recurrence-free survival after the last surgery without adjuvant chemotherapy. The 36 months of recurrence-free survival in our case was the longest survival compared with the reported cases of tumor thrombus in the SMV caused by colon cancer (Table 1). For venous tumor thrombus, even if it is accompanied by liver metastasis, chemotherapy and complete surgical tumor resection may provide long-term survival.

## Conclusion

We encountered a rare case of tumor thrombus in the SMV secondary to colon cancer. Complete surgical tumor resection with chemotherapy is likely to provide long-term survival.

## Data Availability

Not applicable.

## Abbreviations

SMV: Superior mesenteric vein
AST: Aspartate aminotransferase
ALT: Alanine aminotransferase
CT: Computed tomography
PET–CT: Positron emission tomography–computed tomography
CEA: Carcinoembryonic antigen
CA19-9: Cancer antigen 19-9
EGFR: Epidermal growth factor receptor
